# Dietary nitrate attenuates high-fat diet-induced obesity via mechanisms involving higher adipocyte respiration and alterations in inflammatory status

**DOI:** 10.1016/j.redox.2019.101387

**Published:** 2019-11-18

**Authors:** M. Peleli, D.M.S. Ferreira, L. Tarnawski, S. McCann Haworth, L. Xuechen, Z. Zhuge, P.T. Newton, J. Massart, A.S. Chagin, P.S. Olofsson, J.L. Ruas, E. Weitzberg, J.O. Lundberg, M. Carlström

**Affiliations:** a Department of Physiology and Pharmacology, Karolinska Institutet, Stockholm, Sweden; b Department of Medicine, Centre for Molecular Medicine, Karolinska Institutet and Karolinska University Hospital, Stockholm, Sweden; c Department of Molecular Medicine and Surgery, Integrative Physiology, Karolinska Institutet, 17177, Stockholm, Sweden; d Institute for Regenerative Medicine, Sechenov University, Moscow, Russia; e Department of Women's and Children's Health, Karolinska Institutet, Stockholm, Sweden

**Keywords:** Inorganic nitrate, Metabolic syndrome, Mitochondria, Visceral fat, Inflammation, Thermogenesis

## Abstract

Emerging evidence indicates that dietary nitrate can reverse several features of the metabolic syndrome, but the underlying molecular mechanisms still remain elusive. The aim of the present study was to explore mechanisms involved in the effects of dietary nitrate on the metabolic dysfunctions induced by high-fat diet (HFD) in mice. Four weeks old C57BL/6 male mice, exposed to HFD for ten weeks, were characterised by increased body weight, fat content, increased fasting glucose and impaired glucose clearance. All these metabolic abnormalities were significantly attenuated by dietary nitrate. Mechanistically, subcutaneous primary mouse adipocytes exposed to palmitate (PA) and treated with nitrite exhibited higher mitochondrial respiration, increased protein expression of total mitochondrial complexes and elevated gene expression of the thermogenesis gene UCP-1, as well as of the creatine transporter SLC6A8. Finally, dietary nitrate increased the expression of anti-inflammatory markers in visceral fat, plasma and bone marrow-derived macrophages (Arginase-1, Egr-2, IL-10), which was associated with reduction of NADPH oxidase-derived superoxide production in macrophages. In conclusion, dietary nitrate may have therapeutic utility against obesity and associated metabolic complications possibly by increasing adipocyte mitochondrial respiration and by dampening inflammation and oxidative stress.

## Highlights

• Dietary nitrate attenuates high-fat diet-induced adiposity and glucose intolerance.
• Mechanistically, boosting the nitrate-nitrite-NO pathway:
  ○ induces expression of thermogenesis-associated genes in adipocytes
  ○ increases adipocyte respiration
  ○ attenuates macrophage-induced oxidative stress
  ○ modulates inflammatory profile

## Graphical abstract

**Figure:**
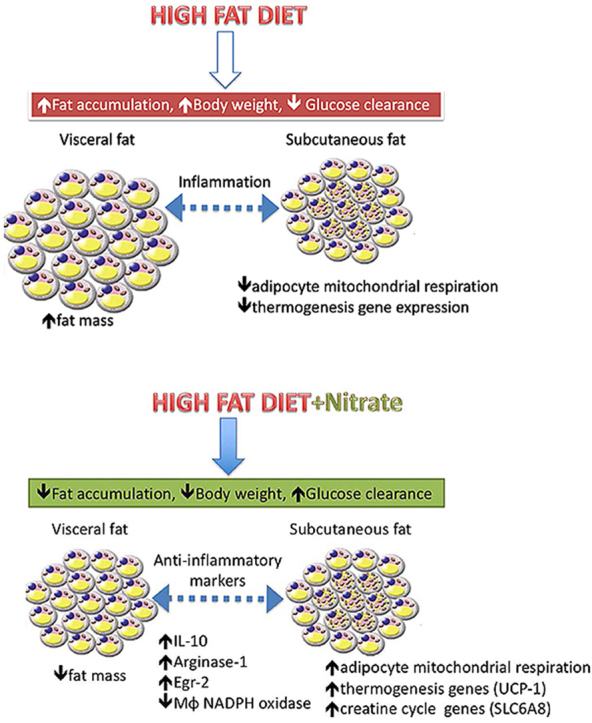

## List of abbreviations

BAT: brown adipose tissue
BSA: Bovine Serum Albumin
CKMT1: creatine kinase, mitochondrial 1
DEXA: dual-emission x-ray absorptiometry
Egr-2: early growth response protein 2
FCCP: carbonyl cyanide-4-(trifluoromethoxy)phenylhydrazone
HFD: high-fat diet
IL-6: interleukin 6
IL-10: interleukin 10
NO: nitric oxide
OCR: oxygen consumption rate
PA: palmitic acid
PGC-1a: peroxisome proliferator-activated receptor gamma coactivator 1-alpha
RER: respiratory exchange ratio
SLC6A8: solute carrier family 6 member 8
UCP-1: uncoupling protein 1
WAT: white adipose tissue

## 1 Introduction

Metabolic syndrome is a combination of metabolic and biochemical abnormalities, often associated with aging and obesity, which increase the risk of developing of type 2 diabetes and cardiovascular disease [1]. Although pharmacological interventions to reduce the risk of type 2 diabetes and pre-diabetic metabolic dysfunctions are available, there is a constant demand for new and more cost-efficient treatments with fewer side-effects [2].

Inorganic nitrate is found in our daily diet and is particularly high in green leafy vegetables, a food group that has been associated with favourable cardiometabolic effects in meta-analyses and systematic reviews [3,4]. After intake, nitrate can be serially converted to nitrite and nitric oxide (NO) as well as other bioactive nitrogen oxide species conveying the effects of this anion [[5], [6], [7]]. Studies conducted over the past couple of decades have shown many favourable cardiovascular effects of dietary nitrate [[5], [6], [7]]. Since originally described in 2010 [8], numerous studies have also showed that supplementation with inorganic nitrate can reverse many features of the metabolic syndrome by restoring nitric oxide (NO) homeostasis in different animal and cellular models [9].

In obesity, lower adipocyte mitochondrial respiration positively correlates to fat accumulation [10]. Additionally, a growing amount of evidence indicates the existence of interactions between innate immunity and adipocyte function, which can influence both metabolism and inflammatory processes [11,12]. Obesity-induced changes of macrophage and adipocyte phenotypes lead to chronic low grade inflammation and insulin resistance, which in turn increase cardiometabolic risk, thereby creating a vicious cycle [13]. Some recent studies indicate that nitrate can increase adipocyte respiration and fatty acid oxidation [14], and reduce infiltration of macrophages in atheromatic plaques of apolipoprotein E−deficient mice fed a high-fat diet (HFD) [15]. The current study aimed at investigating the metabolic effects of the nitrate-nitrite-NO pathway, with specific focus on white adipocyte respiration, inflammatory status and oxidative stress.

## 2 Material and methods

### 2.1 Animals & experimental design

**Fig. 1:**
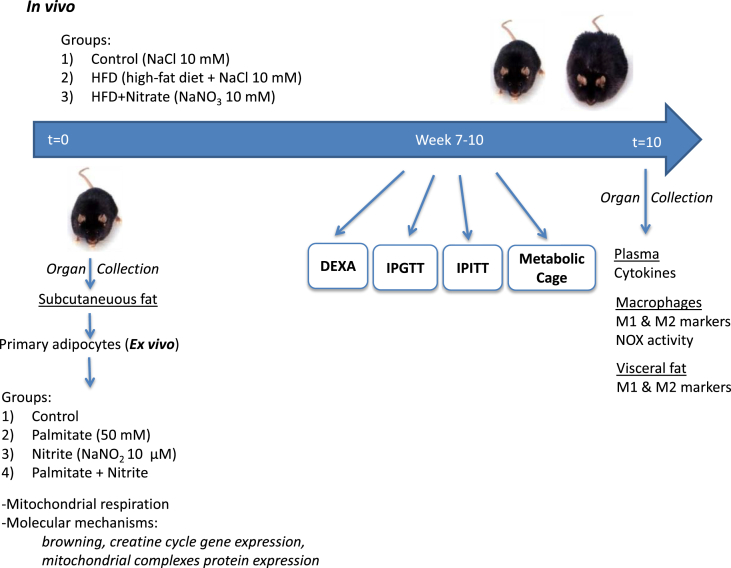
Schematic illustration of the experimental protocol: C57BL/6J (4 weeks old, male) were randomized and divided in 3 experimental groups: A) Control (normal chow, R36, Lantmännen, Sweden, and 10 mM NaCl in the drinking water), B) HFD (Fat 60% kcal, D12492, Research Diets, Inc. NJ, USA) and drinking water supplemented with 10 mM NaCl, C) HFD + Nitrate and drinking water supplemented with 10 mM NaNO_3_. The mice were treated for a total period of 10 weeks and body weight, food and water consumption were monitored on a weekly basis. In vivo measurements of metabolic functions (*i.e.* IPGTT, IPITT, DEXA scanning, physical activity with metabolic cages) were conducted between week 7 and 10 of treatment. After completed protocol the mice were euthanized and organs collected for analyses.

Male C57BL/6J mice (4 weeks old) were obtained from Janvier laboratories (France) and housed under light-dark, temperature and humidity-controlled environment with free access to standard rodent chow and tap water. All animal procedures were approved by the Stockholm Ethical Committee for Animal Experiments (Protocols: N139/15, N164/16) and were carried out in accordance with the EU Directive 2010/63/EU for animal experiments. Fig. 1 gives an overview of the experimental protocol used in this study. In brief, mice were randomized into 3 experimental groups, *i.e.* Control, HFD and HFD + Nitrate supplementation. The mice were treated for 10 weeks and body weight, food and water intake were continuously monitored, and intraperitoneal insulin and glucose tolerance tests, as well as whole-body composition and metabolism were measured between week 7–10, and subsequently sacrificed for organ collection and analyses. From controls and mice chronically treated with nitrate alone for 7 weeks, inguinal fat depots were isolated and used for analysis of mitochondria, browning, fatty acids metabolism and glucose metabolism related gene expression.

### 2.2 Intraperitoneal glucose and insulin tolerance tests

Intraperitoneal Glucose and Insulin tolerance tests were performed as previously described [16]. In brief, mice were injected with 30% d-glucose (2 g/kg body weight) or with insulin (0.75 IU/kg body weight) solutions and repeated blood samples were collected during 120 min after injection.

### 2.3 Body composition analysis

Dual-emission x-ray absorptiometry (DEXA) studies were performed using a Lunar PIXImus densitometer (GE Medical-Lunar, Madison, WI, USA) in isoflurane-anaesthetised (Forene; Abbott Scandinavia AB, Solna, Sweden) animals to determine fat and lean masses, as previously described [17].

### 2.4 Analysis of resting energy metabolism and physical activity

Oxygen consumption, carbon dioxide production, and respiratory exchange ratio (RER: VO_2_/VCO_2_), food and water consumption and locomotion were measured for 24 h by single housing the mice in metabolic cages as previously described [18]. Mice were allowed to first acclimate in the metabolic cages for 24 h followed by subsequent measurements for the next 24 h.

### 2.5 Plasma cytokines

The inflammatory cytokines in plasma were detected using mouse proinflammatory 7-Plex Ultra-sensitive Kit from MesoScale Discovery (MSD, Rockville, MD, USA) following manufacture instructions and as described previously [19].

### 2.6 Isolation and culture of bone marrow macrophages

Bone marrow macrophages were harvested from the femurs of mice derived from the 3 different treatment groups (Control, HFD, HFD + Nitrate, bones were collected from at least 3 different animals/treatment group) as previously described [19].

### 2.7 Inguinal primary adipocytes and cell treatments

Mouse primary adipocytes from inguinal fat depots of 4 week-old male mice were isolated and prepared as previously described [20]. A differentiation cocktail was used during the first 4 days of culture, followed by T3 and insulin for up to 8 days. After differentiation, cells were treated with or without 50 mM palmitate conjugated with bovine serum albumin (BSA) and with or without nitrite (NaNO_2_, 10 μM).

### 2.8 Cellular respirometry by extracellular flux analysis

Mitochondrial respiration was assessed in fully differentiated white mouse primary adipocytes treated as described above. Oxygen consumption rate (OCR) was measured using the Seahorse® XF 24 analyzer (Agilent). The analysis was performed in Dulbecco's Modified Eagle's Medium pH 7.4, 25 mM glucose, and 1 mM pyruvate as substrate. The oxygen consumption rate (OCR) was measured at baseline and followed by sequential stimulation with oligomycin (1 μM), carbonyl cyanide-4-(trifluoromethoxy)phenylhydrazone (FCCP, 1 μM), and antimycin A (2 μM). Experiments were repeated a minimum of 4 times and normalized by cell number.

### 2.9 RNA extraction and gene expression analysis

Total RNA from white mouse primary adipocytes, bone marrow-derived macrophages and visceral fat was extracted by using a combined protocol with Trizol and the columns of the RNeasy mini kit (QIAGEN, Sollentuna, Sweden) as previously described [21]. mRNA was subsequently reversely transcribed to cDNA using the High-Capacity RNA-to-cDNA Kit (Life Technologies, Sweden) and gene expression was analysed with Real-Time PCR using a SYBR Green Master Mix for the primary adipocytes (Life Technologies, Sweden), and TaqManTM Universal PCR Master Mix for the analysis of M1 and M2 markers in visceral fat and in bone marrow-derived macrophages. Detailed sequences of primers and TaqMan hydrolysis probes are in Supplement (Tables S1 and S2).

### 2.10 NADPH oxidase activity in bone marrow macrophages

Both non-treated bone marrow macrophages and macrophages activated with lipopolysaccharide (LPS) from *Escherichia coli* endotoxin (*i.e.* over-night incubation) were used in this study. NADPH oxidase-mediated superoxide production was assessed with lucigenin-derived chemiluminescence as previously described [22]. Briefly, bone marrow macrophages plated on 12-well plates were harvested in PBS and NADPH oxidase-mediated superoxide production was measured in the presence of NADPH (Sigma, 100 μΜ) and lucigenin (Sigma, 5 μΜ) with a Berthold Tubemaster (Autolumat) luminometer.

### 2.11 Western blot analysis of mitochondrial complexes

Cell extracts were lysed in RIPA buffer (50 mM Tris pH 7.5; 150 mM NaCl; 1 mM EDTA; 1% (w/v) TritonX‐100; 0.5% (w/v) Na‐deoxycholate; 0.1% (w/v) sodium dodecyl sulfate (SDS); 20 mM glycerol‐2‐phosphate; 5 mM sodium pyrophosphate) supplemented with phosphatase and protease inhibitors and total protein was extracted. Protein concentration was determined via the Bradford Protein Assay, according to manufacturer's instructions (BioRad). 25 μg of protein was separated via SDS polyacrylamide gel membranes (PAGE) and transferred to PVDF membranes. After blocking with 5% skimmed milk, blots were incubated overnight at 4 °C in Total Oxphos cocktail primary antibody cocktail (ab110413, Abcam) diluted in 0.1% bovine serum albumin (BSA). After washing, membranes were incubated with horseradish-peroxidase-conjugated secondary antibodies for 1 h at room temperature. Blots were visualized by enhanced chemiluminiscence (GE Healthcare) and exposed on Chemidoc (BioRad). Pixels from each band were quantified to measure the individual expression of each complex or the total pixels from each lane were quantified to measure the total Oxphos complexes. All values were normalized the values with GAPDH (Glyceraldehyde-3-phosphate dehydrogenase; MA5-15738-HRP; ThermoFisher).

### 2.12 Cell viability and cell number measurement

Following treatment, viability of cells was assessed via both Trypan blue (0.4%, Gibco®) and Prestoblue (Thermofisher®) assays as previously described [23].

### 2.13 Statistical analysis

Single comparisons between two groups were tested for significance using the Student's paired or unpaired *t*-test as appropriate. Single comparisons between two groups, which did not follow Gaussian distribution were analysed with the non-parametric Mann-Whitney test. Multiple comparisons among groups were analysed by one-way ANOVA followed by recommended post-hoc test. All statistical calculations were made using Graphpad Prism (6.0b, La Jolla, CA, USA). Values are presented as means ± SEM and statistical significance is denoted as p < 0.05.

## 3 Results and discussion

### 3.1 Dietary nitrate attenuates gain in body weight, reduces fat accumulation and improves glucose clearance in mice chronically fed a high-fat diet

**Fig. 2:**
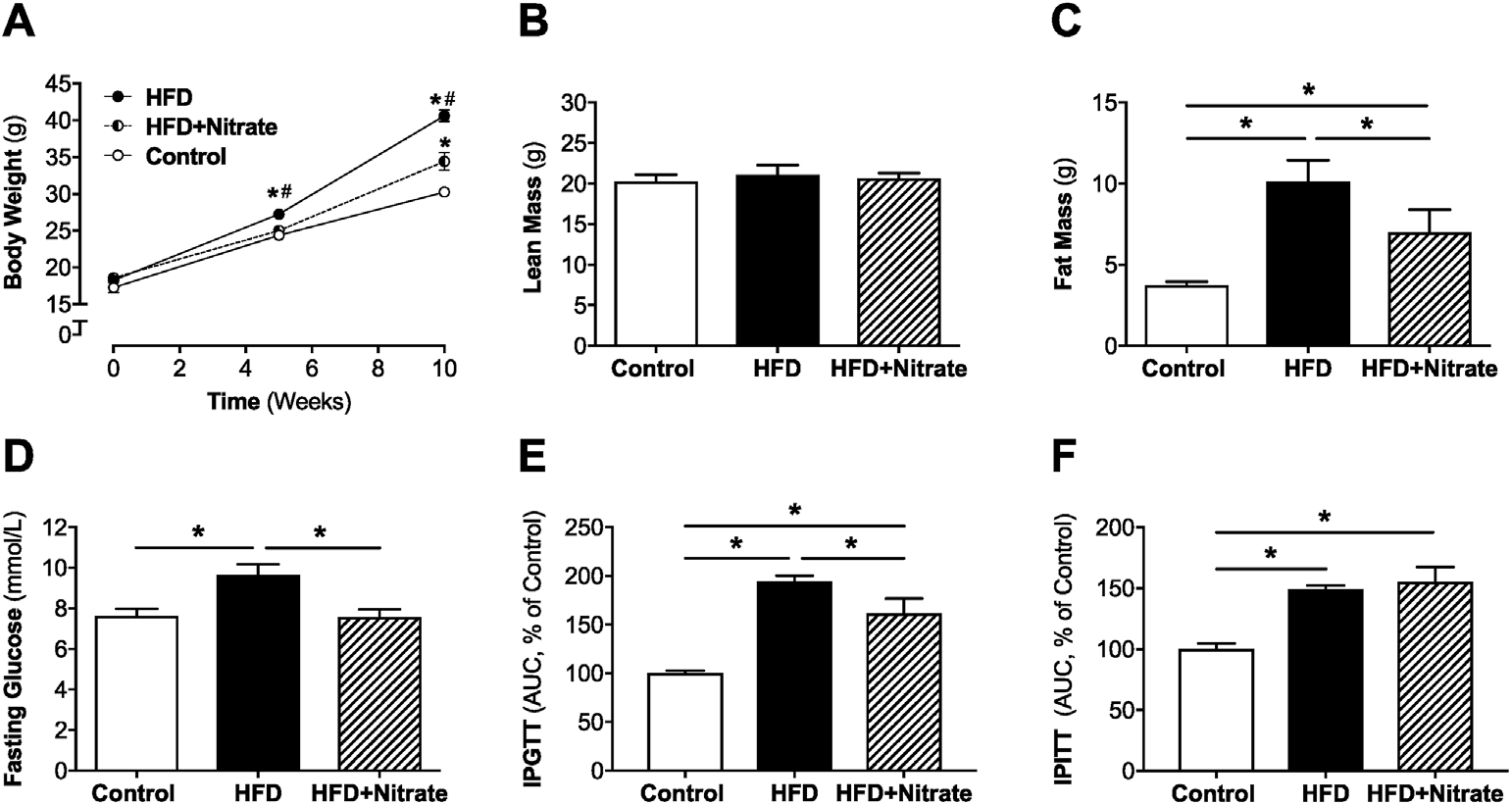
Body weight increase over time (A), Lean and Fat Mass (B, C), Fasting Glucose (D), Intraperitoneal glucose (E) and insulin (F) tolerance tests in Control mice and mice treated with HFD and HFD + Nitrate for 10 weeks. The total Area Under the Curve (AUC) (mmol/L/min) for the 0–120 min period was calculated for the Glucose (E) and Insulin (F) tolerance tests and then expressed as the % compared to controls. Values are shown as mean ± SEM, *n* = 12/group, In panel A *denotes *p* < 0.05 vs time 0 within the same group and #p < 0.05 vs HFD + Nitrate. In panels C–F *denotes *p* < 0.05 vs indicated group.

As expected, HFD progressively increased body weight compared with control mice on normal chow, which was due to accumulation of fat mass (Fig. 2A–C). Moreover, HFD treatment was associated with increased fasting glucose and impaired glucose- and insulin responses (Fig. 2D–F). Nitrate-treated mice did not show changes in locomotion when measured in the metabolic cages compared to HFD alone (Figs. S1A–F). However, increases in body weight and fat accumulation were significantly attenuated (Fig. 2A, C). In addition, HFD-fed mice supplemented with nitrate had no change in response to insulin but had improved glucose homeostasis (Fig. 2D–F), as evident from intraperitoneal insulin and glucose tolerance tests. Taken together, these data suggest that less fat accumulation may not be responsible for the improved glucose clearance observed in nitrate supplemented mice.

Separate analysis of inguinal fat depots isolated from a subset of mice fed normal chow, with or without nitrate in the drinking water for 7 weeks, suggested that some genes associated with mitochondrial function (*CPT1a*), browning (i.e. *UCP1*, *TMEM26*), fat metabolism (*cidea*, *ERRα*), glucose metabolism or non-shivering thermogenesis (*PDK4*, *UCP2*) were upregulated by nitrate supplementation, whereas no significant differences were observed for genes associated with creatine phosphate cycle related genes (Fig. S2 A-E). Overall, these findings are in agreement with previously published studies indicating that nitrate can induce browning and mitochondrial biogenesis [14]. However, changes in mRNA levels do not always correspond with similar changes in protein or activity levels and future in-depth studies are therefore warranted, especially during HFD conditions.

### 3.2 Inorganic nitrite markedly improves mitochondrial respiration in primary adipocytes treated with palmitate

HFD *per se* impairs mitochondrial function and decreases fatty acid oxidation, which leads to excess of adiposity and associated pathologies [24]. Since mitochondria are major targets of inorganic nitrate and nitrite [25,26] and, at least in vascular smooth muscle cells exposed to hypoxia, nitrite stimulates mitochondrial biogenesis and mitochondrial respiration [27], we decided to test the effects of nitrite on the mitochondria of HFD-treated adipocytes. The nitrate-nitrite-NO pathway exerts the majority of its physiological functions via NO-dependent mechanisms [7,9]. The first reduction step of nitrate to nitrite relies mostly on bacterial nitrate reductases present in the oral cavity or in the gut, which is difficult to mimic in the *in vitro* cellular system [7]. Therefore, mouse primary adipocytes were directly treated with nitrite to bypass the first reduction step that normally takes place *in vivo*.

**Fig. 3:**
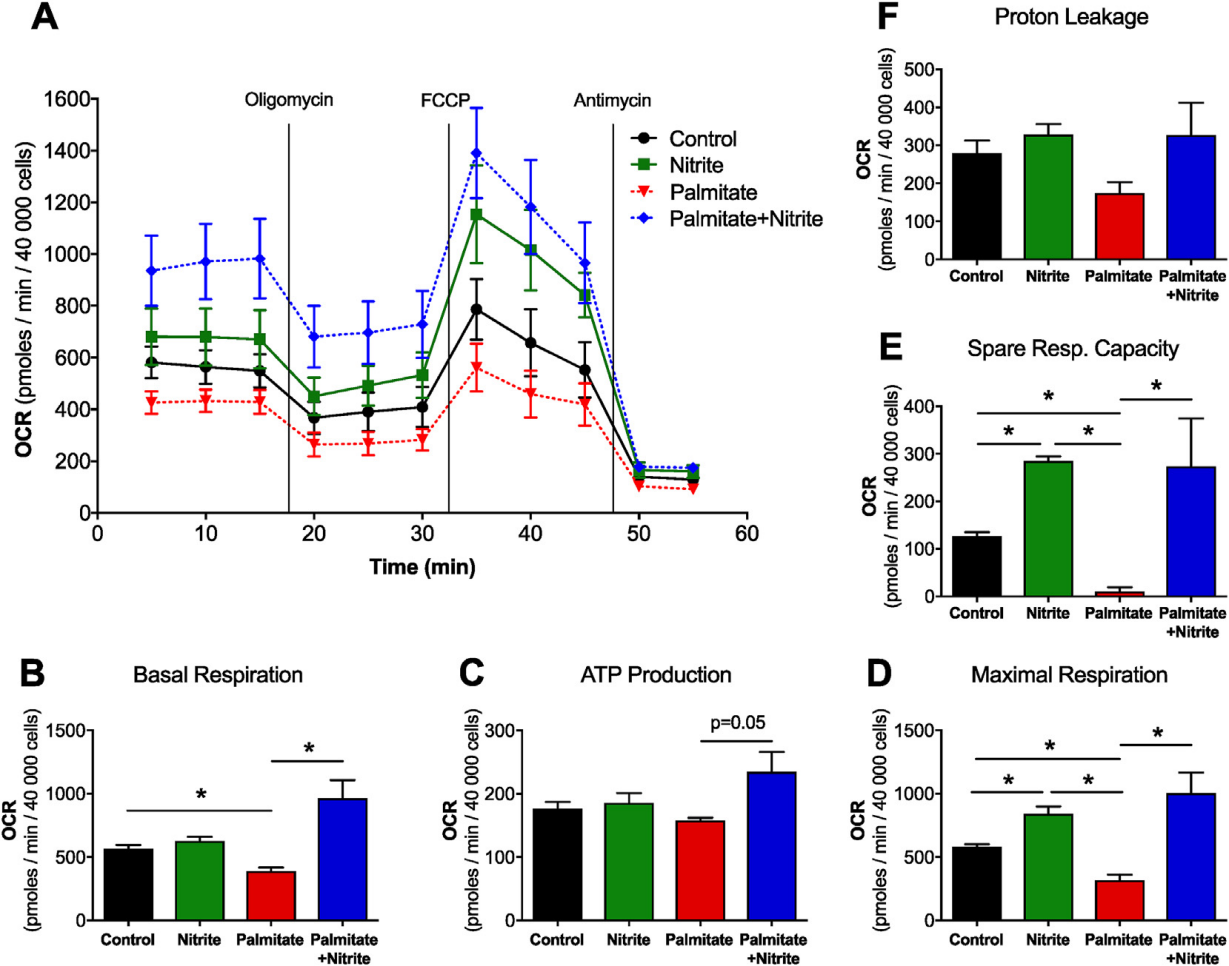
Diagram of the Oxygen consumption rate (OCR) over time (A) measured at baseline and followed by sequential stimulation with oligomycin (1 μM), carbonyl cyanide-4-(trifluoromethoxy)phenylhydrazone (FCCP, 1 μM), and antimycin A (2 μM). Basal and Maximal Respiration (B, D), ATP production (C), Proton leakage (F) and Spare Respiratory Capacity (E) of mouse primary adipocytes treated with Nitrite (10 μΜ), Palmitate (50 mM) or Palmitate + Nitrite for 24 h. Values are shown as mean ± SEM, *n* = 6/group, *p < 0.05 vs indicated group.

To mimic HFD conditions *in vitro* we treated adipocytes with palmitate (PA) conjugated with BSA, as previously described [28]. Compared to control cells PA treatment reduced maximal and basal respiration, proton leak, ATP-induced respiration and spare respiratory capacity (Fig. 3A–F). Nitrite reversed these changes back to control levels or greater. Nitrite also had effects on adipocytes not subjected to PA with increased maximal respiration and spare respiratory capacity (Fig. 3D and E). This is in line with a recent study that indicated that inorganic nitrite can increase basal and maximal mitochondrial respiration in white primary adipocytes [14], however to the best of our knowledge no such study exists in conditions that resemble treatment with HFD.

### 3.3 Inorganic nitrite increases the expression of mitochondrial respiration complexes and thermogenesis genes

**Fig. 4:**
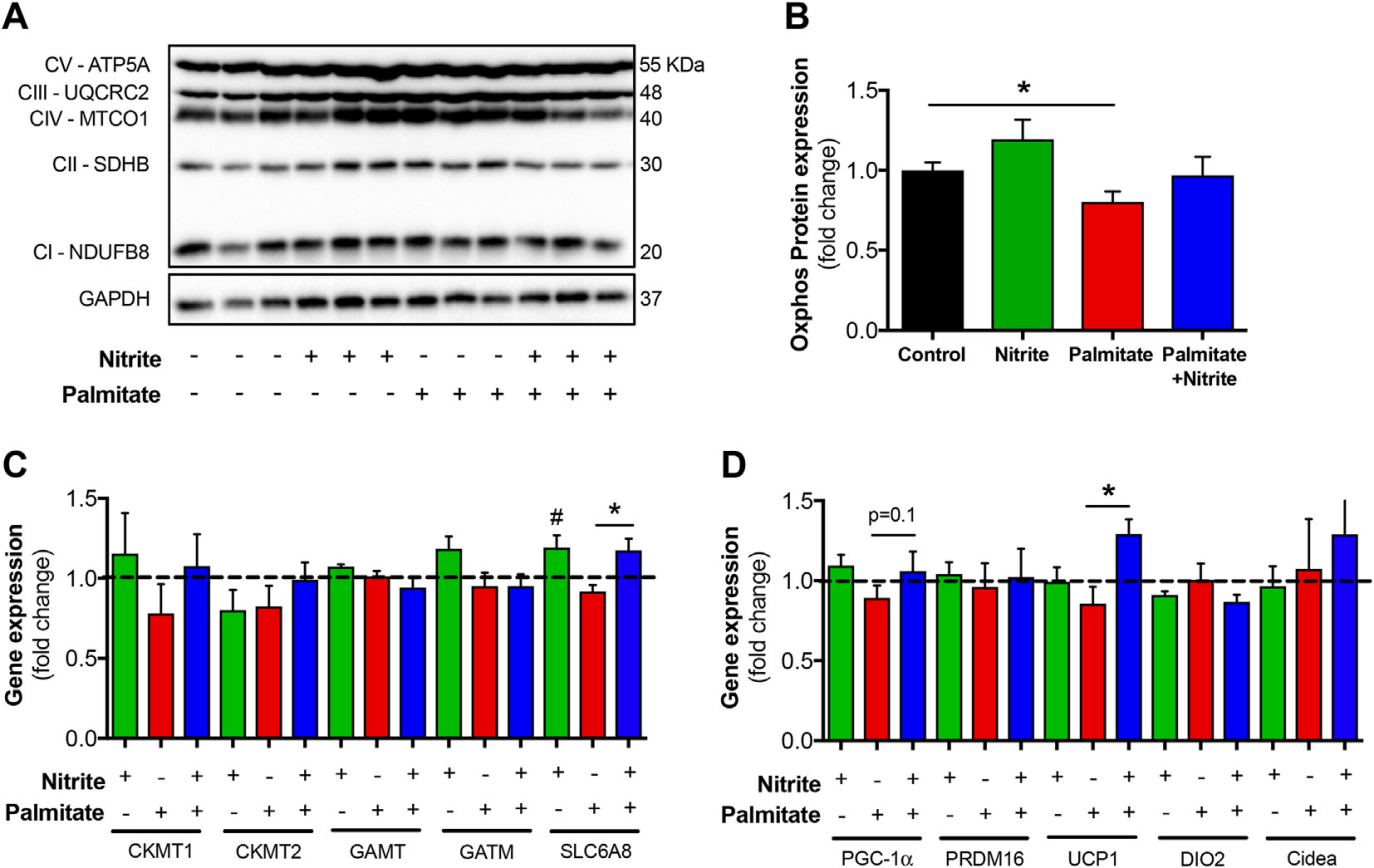
Total mitochondrial complexes protein expression in mouse primary white adipocytes (A, B). Uncropped gels are shown in Fig. S3 together with analysis of the individual mitochondrial complexes (Fig. S4). Expression profiles of genes associated with the creatine cycle (C) and thermogenesis (D) in mouse primary white adipocytes. Dashed line represents naïve adipocytes without any treatment (*i.e.* Control). Cells without any treatment (Control, n = 13) or cells treated with Nitrite (10 μΜ, n = 12), Palmitate (50 mM n = 12) or Palmitate + Nitrite (n = 12) for 24 h. (B), *n* = 4/group (C–D). Values are shown as mean ± SEM. *p < 0.05 vs indicated group, #p < 0.05 vs untreated control.

The coactivator PGC-1α is a master regulator of fatty acid β-oxidation and induces thermogenesis in white adipose tissue (WAT) [29]. UCP-1 is a downstream target of PGC-1α and functions as an inner mitochondrial membrane protein which uncouples proton gradient from ATP production [30], thus resulting in enhanced fatty acid oxidation and thermogenesis in adipocytes [31]. The creatine phosphate cycle has recently been ascribed to play an important role in thermogenesis in the absence of proton-gradient uncoupling [32]. SLC6A8 is a creatine transporter in adipocytes and a recent study has shown that ablation of adipocyte creatine transport impairs thermogenesis and induces obesity [33]. We observed that PA reduces the total protein expression of mitochondrial complexes (Fig. 4A and B and Figs. S3–S4), which can be partially restored by nitrite treatment. Interestingly, nitrite significantly up-regulated gene expression levels of UCP-1 and SLC6A8 (Fig. 4C and D) during PA treatment. Taken together, this indicates nitrite-induced thermogenesis via mechanisms which are both dependent and independent of proton-gradient uncoupling. Since nitrite did not alter cell viability (Fig. S5), it suggests that nitrite may promote mitochondrial biogenesis and browning of WAT, which in turn could explain the enhanced mitochondrial respiration (Fig. 3A) and the attenuated gain in body weight (Fig. 2A). Induction of UCP-1 has been associated with reduced mitochondrial ROS in BAT and alterations of innate immunity [34], however nothing is known about a potential link between these two and browning of adipocytes. Thus, in future studies it would be interesting to investigate whether this nitrite-triggered induction of UCP-1 can modify mitochondrial ROS levels, considering that nitrate and nitrite have a major role in dampening ROS production [35].

### 3.4 Dietary nitrate up-regulates IL-10 and IL-6, and reduces oxidative stress in macrophages of HFD fed mice

**Fig. 5:**
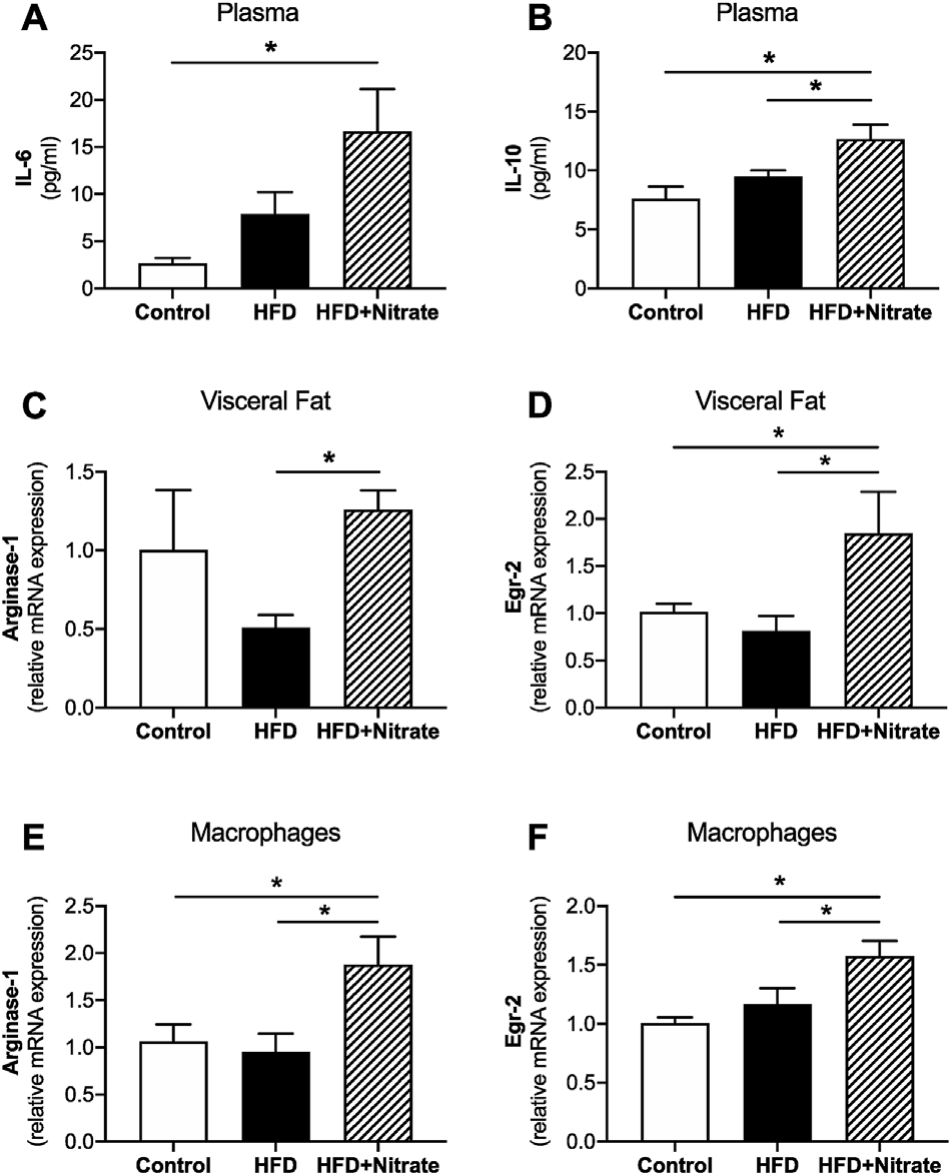
Plasma levels of IL-6 (A) and IL-10 (B), Arginase-1 and Egr2 mRNA expression in visceral fat (C, D) and bone marrow-derived macrophages (E, F), relative to control. Cells, plasma and visceral fat were obtained from control, HFD and HFD + Nitrate treated mice as described in the method section. Values are shown as mean ± SEM, *n* = 6/group (A–E) and n = 9/group (F). *p < 0.05 vs. indicated group.

The pathophysiology of obesity has been linked to inflammatory changes in the circulation coupled with infiltration of immune cells in adipose tissue and alteration of their phenotype [24]. Mice fed HFD and dietary nitrate displayed increased plasma levels of the anti-inflammatory cytokine IL-10 as well as increased levels of IL-6 (Fig. 5A-B). Interestingly, IL-6 had previously been classified as a proinflammatory cytokine but several studies show also anti-inflammatory properties and that increased secretion of IL-6 into the circulation is linked to favourable metabolic effects that are mediated via mechanisms that include beiging of WAT and improved insulin sensitivity [[36], [37], [38]].

**Fig. 6:**
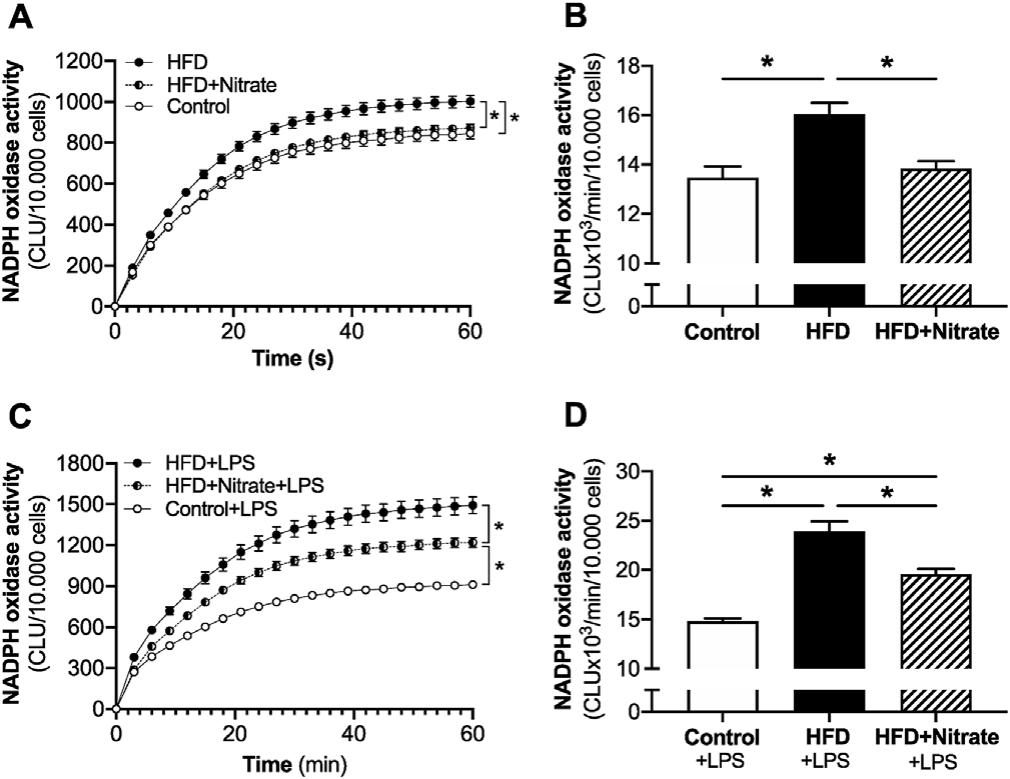
NADPH oxidase activity measured by lucigenin-dependent chemiluminescence of superoxide every 3 s for 1 min in bone marrow-derived macrophages from mice fed normal chow (Control), high-fat diet (HFD) and HFD combined with nitrate treatment (HFD + Nitrate) (A–B). NADPH oxidase activity in macrophages from the same experimental groups following 24 h activation with LPS. Values are shown as mean ± SEM, n = 9/group. *p < 0.05 vs. indicated group.

These observations motivated us to further investigate potential changes in visceral fat, a known target for infiltration and accumulation of immune cells in obesity, and in bone marrow-derived macrophages from the *in vivo* treated mice. As shown in Fig. 5C–F, nitrate up-regulates the expression of typical M2 anti-inflammatory markers such as Arginase-1 and Egr-2 in both visceral fat and in macrophages, without having any significant impact on M1 markers *(data not shown).* Next, we measured NADPH oxidase activation (*i.e.* NADPH-mediated superoxide production), which is a hallmark of the pro-inflammatory action of macrophages [39]. HFD treatment was associated with significant increase in NADPH oxidase activity in isolated and differentiated macrophages, which was fully reversed by simultaneous dietray nitrate treatment (Fig. 6A and B). Upon activation with LPS, macrophages from mice with HFD displayed markedly increased NADPH oxidase activity compared with LPS treated Control macrophages, which again was significantly attenuated by dietary nitrate (Fig. 6C and D).

Studies from our group and by others, have demonstrated that dietary supplementation with inorganic nitrate, to boost the nitrate-nitrite-NO pathway, is coupled with potent anti-inflammatory and antioxidative effects both *in vitro* and *in vivo* [15,19,22,40]. Mechanistically, we have previously showed that inorganic nitrite can attenuate NADPH oxidase-derived superoxide generation in activated peritoneal macrophages and human monocytes [22,41]. However, we believe that this is the first study emphasizing that dietary nitrate has anti-inflammatory effects via modulation of macrophage phenotype in a mouse model of diet-induced obesity and metabolic dysfunction. Nguyen et al. elegantly demonstrated that M2 macrophages upregulate the expression of thermogenic genes and promote fatty acid mobilisation in WAT [42]. Our findings demonstrating nitrate-mediated upregulation of M2 markers might therefore also be linked to the observed thermogenic effects, however future studies are warranted to further explore this possibility. Finally, it is important to clarify that the molecular alterations observed by dietary nitrate treatment in the current study may not be causal of the favourable changes seen in body fat accumulation or glucose metabolism. In particular, the effects of nitrite observed in culture might not necessarily be responsible for the improved glucose clearance seen *in vivo*. It is possible that other mechanisms are responsible for the effects of nitrate, which then result in *e.g.* attenuation of inflammatory signalling and protection of mitochondrial function.

## 4 Conclusions and perspectives

Our findings suggest that dietary supplementation with nitrate may protect against diet-induced obesity via mechanisms that include increased adipocyte mitochondrial respiration, modulation of immune cell function and dampening of oxidative stress. The current study indicate that nitrate-treatment alone can influence several genes related to mitochondrial function and metabolism, however, this should be carefully investigated at different stages of HFD-induced obesity and metabolic disease. Moreover, to further understand the proposed anti-obesity effects of nitrate, future studies are warranted to investigate the possibility that boosting the nitrate-nitrite-NO pathway may affect nutrient absorption during HFD conditions, potentially through alterations in the gut microbiome and modulation of the gut barrier function, which has been suggested previously [43,44]. Current knowledge on inorganic nitrate and modulation of metabolic functions in health and disease has largely been obtained from experimental animal and cell studies, and even though controversies exist (For example [45]) the majority of these studies have demonstrated favourable metabolic effects (See Review [9]). Although more efforts are needed to explain similarities and differences among experimental studies, it will be even more important to investigate the potential therapeutic metabolic effects of dietary nitrate in human trials. Nevertheless, dietary approaches to boost the nitrate-nitrite-NO pathway may have great potential to prevent metabolic syndrome and associated complications.

## Declaration of competing interest

J.O.L and E.W. are co-inventors on patent applications related to the therapeutic use of inorganic nitrate. The other authors have no conflicts of interest.
