# Supplementary material for: Dietary nitrate attenuates high-fat diet-induced obesity via mechanisms involving higher adipocyte respiration and alterations in inflammatory status
Source: Redox Biol. 2019 Nov 18;28:101387. doi: 10.1016/j.redox.2019.101387 (PMC6883295; doi:10.1016/j.redox.2019.101387)
Supplement: Multimedia component 1 [file mmc1.docx]

**Dietary nitrate attenuates high-fat diet-induced obesity via mechanisms involving higher adipocyte respiration and alterations in inflammatory status**

Peleli M^1,*^, Ferreira DMS^1,*^, Tarnawski L^2^, McCann Haworth S^1^, Xuechen L^1^, Zhuge Z^1^, Newton PT^1^, Massart J^3^, Chagin AS^1,4^, Olofsson PS^2^, Ruas JL^1^, Weitzberg E^1,5^, Lundberg JO^1^, Carlström M^1,#^

^1^ Department of Physiology and Pharmacology, Karolinska Institutet, Stockholm, Sweden

^2^ Department of Medicine, Centre for Molecular Medicine, Karolinska Institutet and Karolinska University Hospital, Stockholm, Sweden

^3^ Department of Molecular Medicine and Surgery, Integrative Physiology, Karolinska Institutet, 17177 Stockholm, Sweden

^4^ Institute for Regenerative Medicine, Sechenov University, Moscow, Russia

^5^ Department of Women’s and Children’s Health, Karolinska Institutet, Stockholm, Sweden

**TABLE S1.** *TAQMAN Hydrolysis probes used for the analysis of M1 & M2 markers.*

| Mm03024075_m1 | Hprt |
| --- | --- |
| Mm00475988_m1 | Arg1 |
| Mm00456650_m1 | Egr2 |

**TABLE S2.** *Primer sequences used for the investigation of thermogenesis and creatine cycle gene expression.*

| **Primer** | **Sequence** |
| --- | --- |
| Cidea FW | TGC TCT TCT GTA TCG CCC AGT |
| Cidea RV | GCC GTG TTA AGG AAT CTG CTG |
| Ckmt1 FW | TGA GGA GAC CTA TGA GGT ATT TGC |
| Ckmt1 RV | TCA TCA AAG TAG CCA GAA CGG A |
| Ckmt2 FW | CCA GTG CCT TCT CAA AGT TGC |
| Ckmt2 RV | AGT CCG CAC TTG GGG GAA AGA G |
| DIO2 FW | AAT TAT GCC TCG GAG AAG ACC G |
| DIO2 RV | GGC AGT TGC CTA GTG AAA GGT |
| Gamt FW | GCA GCC ACA TAA GGT TGT TCC |
| Gamt RV | CTC TTC AGA CAG CGG GTA CG |
| Gatm FW | GAC CTG GTC TTG TGC TCT CC |
| Gatm RV | GGG ATG ACT GGT GTT GGA GG |
| PGC-1a_Total FW | TGA TGT GAA TGA CTT GGA TAC AGA CA |
| PGC-1a_Total RV | GCT CAT TGT TGT ACT GGT TGG ATA TG |
| PRDM16 FW | CAG CAC GGT GAA GCC ATT C |
| PRDM16 RV | GCG TGC ATC CGC TTG TG |
| Slc6a8 FW | GTG TGG AGA TCT TCC GCC AT |
| Slc6a8 RV | CCC GTG GAG AGC CTC AAT AC |
| UCP1 FW | CAA TGA ACA CTG CCA CAC CTC |
| UCP1 RV | GGC ATT CAG AGG CAA ATC AGC T |
| HPRT FW | AGT CCC AGC GTC GTG ATT AG |
| HPRT RV | TTT CCA AAT CCT CGG CAT AAT GA |
| TFAM FW | GAGCGTGCTAAAAGCACTGG |
| TFAM RV | ACTTCGGAATACAGACAAGACTGA |
| CPT1a FW | CACTGCAGCTCGCACATTAC |
| CPT1a RV | CCAGCACAAAGTTGCAGGAC |
| PDK4 FW | AGG GAG GTC GAG CTG TTC TC |
| PDK4 RV | GGA GTG TTC ACT AAG CGG TCA |
| TMEM26 FW | ACCCTGTCATCCCACAGAG |
| TMEM26 RV | TGTTTGGTGGAGTCCTAAGGTC |
| TBX1 FW | GGCAGGCAGACGAATGTTC |
| TBX1 RV | TTGTCATCTACGGGCACAAAG |
| UCP2 FW | ATGGTTGGTTTCAAGGCCACA |
| UCP2 RV | CGGTATCCAGAGGGAAAGTGAT |
| ERRa FW | GGGGAGCATCGAGTACAGC |
| ERRa RV | AGACGCACACCCTCCTTGA |
| Cyt C FW | ACAAGAAGACTCAAATGTGTTTCAGTTT |
| Cyt C RV | TGCACTGTCAAGAATAGACAGTTGC |
| GLUT4 FW | AAAAGTGCCTGAAACCAGAG |
| GLUT4 RV | TCACCTCCTGCTCTAAAAGG |
| FABP4 FW | AAGGTGAAGAGCATCATAACCCT |
| FABP4 RV | TCACGCCTTTCATAACACATTCC |

**FIGURE S1**

Measurement of the VO_2_-oxygen consumption (A), VCO_2_-carbon dioxide production (B), RER-Respiratory Exchange Ratio (C), physical activity in the X and Y axis (total number of counts), food (E) and water (F) intake, in conscious mice using metabolic cages. The mice acclimatized in the new environment (i.e. metabolic cages) for 24 h, followed by subsequent measurements for the next 48 h. Averaged data are presented as Mean±SEM, n=6/group, *p<0.05.

**FIGURE S2**

Gene expression in subcutaneous fat obtained from Control or Nitrate treated mice as described in the method section. (A) mRNA expression of mitochondria related genes: PGC-1α (Peroxisome proliferator-activated receptor gamma coactivator 1-α); Cyt c (cytochrome c); TFAM (Transcription Factor A, Mitochondrial); CPT1a (Carnitine palmitoyltransferase 1a). (B) mRNA expression of browning related genes: UCP1 (uncoupling protein 1); PRDM16 (PR domain containing 16); TMEM26 (Transmembrane Protein 26); TBX1 (T-box transcription factor 1). (C) mRNA expression of fatty acids metabolism related genes: Cidea (Cell death activator); ERRα (Estrogen-related receptor α); FABP4 (Fatty Acid-Binding Protein 4); Dio2 (Deiodinase, Iodothyronine Type II). (D) mRNA expression of glucose metabolism related genes: PDK4 (Pyruvate dehydrogenase lipoamide kinase isozyme 4); GLUT4 (Glucose transporter type 4); UCP2 (uncoupling protein 2). (E) mRNA expression of creatine phosphate cycle related genes: CKMT2 (Creatine Kinase, Mitochondrial 2); GAMT (Guanidinoacetate methyltransferase); GATM (Glycine Amidinotransferase); SLC6A8 (Solute Carrier Family 6 Member 8). Values are shown as mean±SEM, *n*=6/group. **p*<0.05 compared with Control.

**FIGURE S3**

Uncropped gels for Western blot analysis of mitochondrial complexes in mouse primary white adipocytes. Five independent experiments for control cells or cells treated with Nitrite (10 μΜ), Palmitate (50 mM) or Palmitate+Nitrite for 24 hours. n = 12 *per* group

**FIGURE S4**

Mitochondrial complexes protein expression in mouse primary white adipocytes. Cells without any treatment (Control, n=13) or cells treated with Nitrite (10 μΜ, n=12), Palmitate (50 mM n=12) or Palmitate+Nitrite (n=12) for 24 hours. Values are shown as mean±SEM.

**FIGURE S5**

Measurement of cell viability in the experimental conditions used for mouse primary white adipocytes. Cell viability was estimated with the Trypan Blue (A) and Presto Blue (B) methods. The methods were performed in a blinded fashion where the person performing the methods was not aware of the different treatment groups. The Trypan Blue measurements (A) were done by cell counting on a haematocytometer whereas the Presto Blue method (B) is based on measuring the fluorescence intensity emitted from viable cells exposed to resazurin. Values are presented as Mean±SEM, n=12/group.
